# The crucial role of the *Aspergillus fumigatus* siderophore system in interaction with alveolar macrophages

**DOI:** 10.1016/j.micinf.2010.07.005

**Published:** 2010-11

**Authors:** Markus Schrettl, Oumaima Ibrahim-Granet, Sabrina Droin, Michel Huerre, Jean-Paul Latgé, Hubertus Haas

**Affiliations:** a Division of Molecular Biology/Biocenter, Innsbruck Medical University, Fritz-Pregl-Str. 3, 6020 Innsbruck, Austria; b Aspergillus Unit, Institut Pasteur, 25 rue du Dr Roux, 75724 Paris, France; c Histopathology Unit, Institut Pasteur, 25 rue du Dr Roux, 75724 Paris, France

**Keywords:** *Aspergillus fumigatus*, Alveolar macrophage, Aspergillosis, Siderophore, Conidial killing, Inflammation

## Abstract

Iron plays a central role in manifestation of infections for a variety of pathogens. To ensure an adequate supply with iron, *Aspergillus fumigatus* employs extra- and intracellular siderophores (low-molecular mass iron chelators), which are of importance for fungal growth in particular during iron starvation. Here we show that the lack of extracellular siderophores, and especially, the lack of the entire siderophore system cause in immunosuppressed mice in vivo (i) a reduced extracellular growth rate, (ii) a reduced intracellular growth rate in alveolar macrophages, and (iii) an increased susceptibility to conidial growth inhibition by alveolar macrophages. These data underline the crucial role of the fungal siderophore system not only for extracellular growth but also in the interaction with the host immune cells. Moreover, the hyphal growth rate within alveolar macrophages compared to extracellular lavage fluid was significantly decreased indicating that, besides elimination of fungal conidia, inhibition of pathogenic growth is a function of macrophages.

## 1 Introduction

*Aspergillus fumigatus* and *Candida albicans* make up the vast majority of clinical incidences caused by fungal pathogens. Infection by *A. fumigatus* leads to a mortality of up to 90% in immuno compromised hosts [1]. Beside the immune status of a host, only a few characterized virulence attributes have been identified. As iron is essential, its acquisition in vivo is required for virulence of bacteria, yeast and, as recently shown, *A. fumigatus* [2–4].

During invasive disease, *C. albicans* acquires iron *via* reductive iron-assimilation and, possibly, heme uptake [5]. *A. fumigatus* lacks heme utilization [4], employs as *C. albicans* reductive iron-assimilation and additionally siderophore-mediated iron mobilization [6,7]. *A. fumigatus* virulence is absolutely dependent on a functional siderophore system whereas reductive iron-assimilation has limited impact during infection in a mouse model for pulmonary aspergillosis [4]. Siderophores are low-molecular mass ferric-iron specific chelators, the production of which is up-regulated during iron starvation. *A. fumigatus* excretes two major siderophores to capture extracellular iron, namely fusarinine C and its acetylated derivative triacetylfusarinine C. The hyphal siderophore ferricrocin plays an important role in intracellular iron distribution and iron storage, and the conidial siderophore hydroxyferricrocin is of crucial importance for conidial iron storage, proper germination and oxidative stress resistance [8]. Subsequent to uptake by specific transporters [9], iron-loaded siderophores are hydrolyzed by the esterase EstB; the iron is supplied to the metabolism or transferred to ferricrocin [10]. *A. fumigatus* cannot directly utilize the host iron compound transferrin but triacetylfusarinine C has the capability to remove iron from transferrin [4,11].

Subsequent to inhalation, *A. fumigatus* conidia are confronted with alveolar macrophages (AM), which represent the most frequent resident phagocytic cell line in the lung and an essential line of defense against pathogens. Moreover, macrophages play an important role in iron homeostasis of the host [12]. Under inflammatory conditions macrophages are prominent storage sites for iron. Cytokines, acute phase proteins and radicals produced by macrophages modulate iron uptake and iron release in these immune cells. Iron is required to produce highly toxic radicals and at the same time iron regulates cytokine activities and modulates lymphocyte and neutrophil activation and differentiation [13].

We have previously shown that siderophores are important for saprobic growth, particularly during iron starvation, and virulence [14]. A lack of both intra- and extracellular siderophores due to deficiency in the ornithine monooxygenase SidA (*ΔsidA* strain) was shown to render *A. fumigatus* avirulent in a neutropenic mouse model of invasive aspergillosis [4]. Histological analysis indicated that *ΔsidA* conidia do not germinate in vivo; at 60 h postinfection *ΔsidA* conidia are still present in bronchioles with very limited cellular recruitment to foci of infection. *ΔsidC* strains, which lack intracellular siderophores due to deficiency in the nonribosomal peptide synthetase SidC [14], as well as *ΔsidF* and *ΔsidD* strains, which lack extracellular siderophores due to deficiency in the acyl transferase SidF or the nonribosomal peptide synthetase SidD [14], showed attenuated virulence. Histopathology revealed that infection with these mutant strains result in reduced inflammation. Recently, we demonstrated that during in vitro infection with *A. fumigatus sidA*-deficiency changes immune effector pathways and iron homeostasis in murine RAW264.7 macrophages [15]. In the present study we investigated the individual and combined roles of extra- and intracellular siderophores in resistance of *A. fumigatus* against elimination by AM’s ex vivo and in vivo.

## 2 Materials and methods

### 2.1 Fungal strains

Fungal strains (Table 1) were cultured at 37 °C on 2% malt extract agar slants, containing 1.5 mM FeSO_4_ for three days and were then kept on 22 °C until use. For the macrophage experiments conidia were labeled with fluorescein isothiocyanate (FITC) (Sigma). Freshly harvested conidia were filtered through a 40 μm cell strainer (Falcon) and a final concentration of 2 × 10^7^conidia in 10 ml 0.05 M Na-carbonate buffer (pH10.2) were incubated with FITC at a final concentration of 0.1 mg/ml at 37 °C for 1 h and washed three times by centrifugation in PBS-0.1% Tween 20 (PBST) [16,17]. PBST was also used as conidial buffer.

### 2.2 Chemicals and antibodies

FITC, mouse and goat sera, and *p*-formaldehyde were obtained from Sigma. RPMI 1640 containing glutamine and with (RPMI complete) or without heat-inactivated FCS were obtained from GIBCO BRL (Cergy Pontoise, France). A rabbit polyclonal antibody (pAb) specific for *A. fumigatus* conidia was used as a primary antibody [18]. As secondary antibody, a Texas Red-conjugated goat anti-rabbit IgG (Jackson Immunoresearch Laboratory) was used.

### 2.3 Murine infection assay

Murine infections were performed in consistence with the guidelines for animal experiments from the Institute Pasteur in compliance with European animal welfare regulation. For all the studies, 6–8 week old male outbred Swiss OF1 mice weighing approximately 25 g (Iffa Credo, Saint-Germain sur l’Arbresle, France) were used.

Mice were immunosuppressed intraperitoneally with hydrocortisone acetate (25 mg; Sigma, St. Louis, Mo.) at day-3 relative to infection and immediately before intranasal inoculation (day 0). Mice were anesthetized by intramuscular injection of 0.1 ml of a solution containing 10 μg/ml ketamine (Merial) and 2 mg/ml xylasine (Bayer) and infected by intranasal instillation of 2 × 10^6^ FITC-labeled conidiospores in 25 μl PBST. Mice were monitored daily. For histological sections, lungs were removed after lavage washes and fixed in 4% v/v formaldehyde. Lungs were embedded in paraffin prior to sectioning and stained as already described [19]. Lung sections from at least two mice per strain were analyzed regarding the inflammation indices and fungal burden as described earlier [20] (See also Results 3.4). Importantly, each lung was analyzed investigator-blinded at the Histopathology Unit at the Institut Pasteur.

### 2.4 Isolation of AM

AM were harvested from mouse lungs with 10 ml of ice-cold Ca^2+^- and Mg^2+^-free PBS through an 18-gauge plastic catheter inserted into the trachea after cervical dissection. Cells were separated from lavage fluid by centrifugation at 400 g for 10 min at room temperature (RT) and resuspended in 0.5 ml–1 ml RPMI complete. Aliquots of 500 μl, containing 5 × 10^5^ cells, were added to 4-well Permanox slides (Lab-Tek; Nalge Nunc International Corp., Naperville, Ill.). The cells were allowed to adhere for 30 min at 37 °C under a humidified atmosphere with 5% CO_2_. If necessary, cells were washed three times with RPMI 1640 after adherence.

### 2.5 Phagocytosis assay

AM harvested from immuno-competent mice as described above, were incubated with 5 FITC-labeled conidia per macrophage for 30 min at 4 °C. Unbound conidia were removed by washing with cold RPMI complete medium, and phagocytosis of the bound conidia was initiated by shifting the cells to 37 °C in an atmosphere of 5% CO_2_ for 1 h [17]. Phagocytosis was stopped by incubating the cells with 3% p-Formaldehyde for 9 min at RT followed by 50 mM NH_4_Cl for 9 min at RT. Blocking was performed by incubation of the cells in 5% goat serum and 5% mouse serum in PBS (Blocking serum) for 30 min at RT. For specific labeling of uningested conidia, AM were incubated for 30 min at RT with a rabbit anti-conidia antiserum at a dilution of 1:50, washed three times with blocking serum, and finally incubated for 30 min at RT with the Texas Red-conjugated anti-rabbit antibody at a dilution of 1:200. In order to permeabilize cells for nuclear staining with Hoechst 33342 (Molecular Probes, Eugene, Oreg.), AM were incubated with 1% Saponin in blocking serum. After incubation with Hoechst 33342 in 1% Saponin-blocking serum for 10 min at RT, cells were washed; the rate of phagocytosis was estimated as the ratio of the number of ingested conidia to the total number of conidia counted multiplied by 100.

### 2.6 In vivo killing of conidia and determination of hyphal length

After cells were separated from lavage fluid as described above, the conidial killing was quantified after 24 and 48 h. Therefore AM were lysed by a water osmotic shock and after addition of an equal volume of 2 × Sabouraud (4% glucose, 2% mycopeptone) culture medium, the homogenate (containing FITC-labeled conidia and lysed AM) was then incubated at 37 °C for 6 h. Important to note, spherical conidia from *wt* and mutant strains with or without obvious germ tube, generated hyphae in Sabouraud within 6 h. In contrast, distorted and half-moon like shaped conidia failed to germinate and grow in Sabouraud even after prolonged (16 h) incubation at 37 °C. Therefore, the growth rate was routinely scored after 6 h and the failure to grow was defined as killing. The conidial killing was calculated by fluorescence microscopy as the percentage of the number of non-germinated conidia to the total number of conidia (*n* ≥ 300) counted. Notably, conidia of all *A. fumigatus* strains used displayed a germination rate >95% in this medium. Alternatively, the ratio of presumably killed (distorted or half-moon like shaped) versus not-killed conidia (spherical or germinated) was scored immediately after harvesting AMs from the lungs without further incubation by fluorescence microscopy (data not shown). This method yielded essentially the same results as the above assay underlining scoring of killing rather than growth inhibition by the assay used. About 80% of the conidia in the lavage fluid were found within AMs. Distorted or half-moon like shaped conidia were only found within AMs but not extracellularly indicating that killing required AM’s.

To determine the in vivo hyphal growth rate, hyphal length (*n* ≥ 100 hyphae for each strain and time point) was determined in lavage fluids using the MetaVue program with a cool snap camera (Universal Imaging corp.). Statistical data for conidial killing and hyphal length were analyzed by one- and/or two-tailed *T*-test for independent samples.

### 2.7 Fluorescence microscopy

For fluorescence microscopy, samples were examined with a Zeiss Axiophot microscope attached to a cooled charge-coupled device camera (Photometrics).

## 3 Results

### 3.1 Siderophore deficiency does not affect phagocytosis by AM

The phagocytosis rate was determined with AM, which were freshly harvested from immuno-competent mice incubated with conidia at a ratio of 1:5. After 60 min, about 75% of wt, 69% of *ΔsidA* and 70% of *ΔsidF* conidia were internalized by AM (data not shown). Thus, internalization rates with freshly harvested AM are comparable to results obtained with the macrophage cell line RAW264.7 from in vitro experiments [15] and neither the lack of extracellular siderophore biosynthesis or the entire siderophore system alters the phagocytosis rate by AM ex vivo.

### 3.2 Siderophore deficiency increases in vivo killing of conidia

**Fig. 1:**
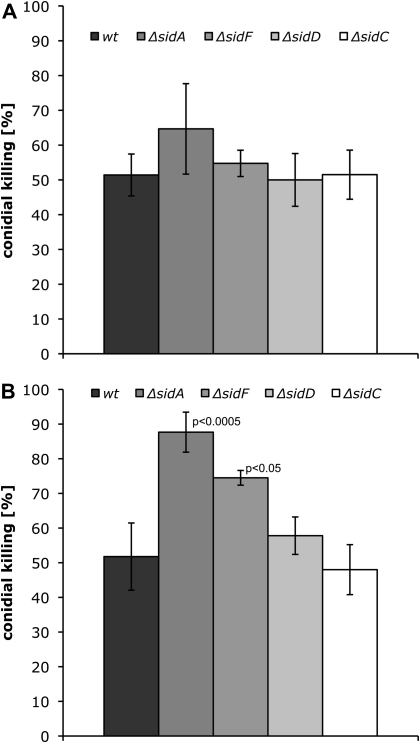
Analysis of in vivo conidial growth inhibition by AMs 24 h (A) and 48 h (B) after infection. *A. fumigatus* conidia were harvested *via* bronchoalveolar lavage and the ability to germinate and grow was determined microscopically. Percentage of non-germinated to the total number of conidia was bar-plotted. Values represent means of three mice per strain per time point including standard deviation. Given *p*-values of *sidA* and *sidF* mutant strains indicate the statistical significance of the difference to *wt*.

Killing of conidia of the different fungal strains by AM was investigated 24 and 48 h after infection of immunosuppressed mice as described in Material and methods. For each strain at either time point three animals were examined. After 24 h, about 50% of conidia of *wt*, *ΔsidF*, *ΔsidD* and *ΔsidC* were killed (Fig. 1A). Killing of *ΔsidA* conidia was slightly (but statistically not significant) increased to 64%. After 48 h, the killing rate for *wt* and *ΔsidC* conidia was still about 50% (Fig. 1B). In contrast, 88% (*p* < 0.0005) of *ΔsidA* and 75% (*p* < 0.05) of *ΔsidF* conidia were killed, respectively. The killing rate of *ΔsidD* conidia was increased to 58%. Importantly, the respective complemented mutant strains (Table 1) behaved like the *wt* (data not shown) underlining that the effects scored are indeed due to the defects in siderophore biosynthesis.

### 3.3 Siderophore-deficiency decreases extra- and intracellular in vivo growth

Deficiency in either extra- or intracellular siderophores decreases the growth rate of *A. fumigatus* in vitro during iron-depleted condition only [14]. To study the impact of siderophores on the in vivo growth rate, the hyphal length of *A. fumigatus wt* and mutant strains was measured in lavage fluids 24 and 48 h after infection.

**Fig. 2:**
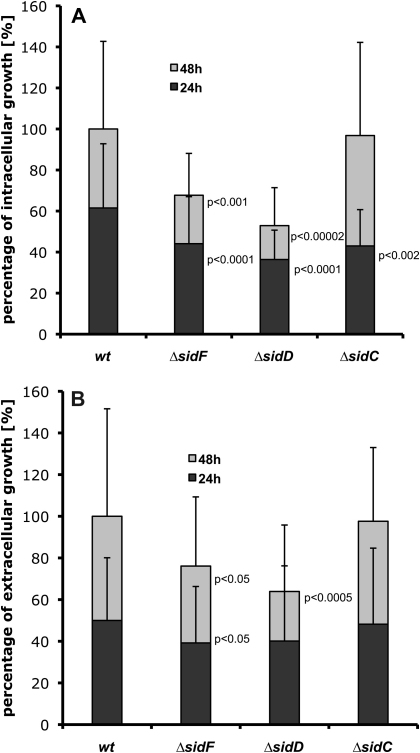
Hyphal length of *A. fumigatus wt*, *ΔsidF*, *ΔsidD*, and *ΔsidC* in vivo. Length of hyphae was determined inside AMs (A) and outside AMs (B). Significant growth reduction is indicated by *p*-values beside the bar. *P*-values were determined using an unpaired, two-tailed *T*-test. Data are derived from three independently infected animals. Error bars represent standard deviations.

Notably, wide differences between hyphal length inside and outside of AMs were found, hence for analysis, hyphae were divided into two groups according to its particular localization inside or outside AMs. Inside AM, *wt* hyphae reached a length of about 6 times the diameter of resting conidia after 24 h and 10 times after 48 h following infection. Outside AM, the *wt* doubled its hyphal length from 16.5 times the conidial diameter after 24 h up to 33 times after 48 h. Compared to *wt*, *ΔsidF* and *ΔsidD* displayed a statistically significant decrease in hyphal length (Fig. 2A) and hyphal growth rate (data not shown) extracellularly and inside of AM after 24 h and 48 h. Remarkably, inside AM *ΔsidC* lagged behind the *wt* after 24 h (*p* < 0.002) but was able to compensate the growth reduction within the following 24 h (Fig. 2A). We have previously shown that *ΔsidC*-caused lack of conidial hydroxyferricrocin delays germination under iron-limiting conditions [14], which most likely explains this delay in hyphal growth of *ΔsidC* after 24 h. Extracellularly, *ΔsidC* behaved like the *wt*. The intra- and extracellular in vivo growth rate of *ΔsidA* hyphae was not determined, since a statistically adequate number of hyphae could not be detected in lavage fluids due to the high killing rate.

### 3.4 Siderophore-deficiency decreases tissue inflammation and invasion

**Fig. 3:**
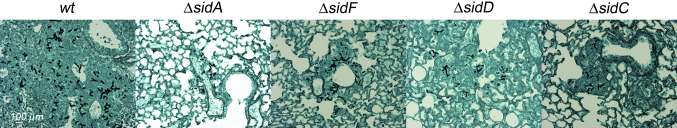
Histopathological analysis of murine lung sections infected with *A. fumigatus*. Sections were prepared after 48 h of infection, fixed in 4% v/v formaldehyde and stained using Grocotts Methenamine Silver.

To compare the differences in growth and survival within AM of the mutant strains with the lung damage caused, the lungs were removed, fixed and sectioned immediately after the lavage fluids were harvested. Sections from at least two mice per strain were analyzed regarding the inflammation indices and fungal burden. The inflammation index was indicated as the percentage of the whole lung surface interspersed with inflammatory foci and subsequently classified between 0 and 5, with 5 describing the most severe lesions (1 = ≤ 20%; 2 = 20–40%; 3 = 40–60%; 4 = 60–80%; 5 = >80%). Similarly, the fungal invasion index was scaled from 0 to 5: 1 = few hyphae around the bronchi; 2 = several foci of hyphae limited to the periphery of the bronchi and blood vessels; 3 = invasive aspergillosis in which the hyphae were observed to cross the vascular wall and extend to the alveoli; 4–5 = severe invasive aspergillosis with massive hyphal invasion resulting in the necrosis of the whole lung. The *wt* displayed an inflammation index of 2 after 24 h of growth in vivo. Some of the sections already showed severe lesions. The fungal invasion index ranged from few hyphae to several foci of hyphae, but no signs of invasive aspergillosis after 24 h. After 48 h the fungal invasion index strongly increased and the majority of the lungs showed clear signs of invasive aspergillosis. Concomitantly, the inflammation index increased up to 3, with most of the lungs showing around 60% inflammatory foci (Fig. 3, Table 2). In stark contrast, *ΔsidA* caused inflammation indices of average 0.5 and a fungal invasion index between 1 and 2, describing only few hyphae and some foci after 48 h (Fig. 3, Table 2). A *ΔsidF*-infection led to moderate lesions, which increased slightly after 48 h. The invasion index stayed relatively constant with some foci of hyphae, but no signs of invasive aspergillosis (Table 2). *ΔsidD*-infected lung tissue showed some single foci of hyphae after 48 h, with little inflammation (Fig. 3, Table 2). Surprisingly, even *ΔsidC* caused only mild lesions after 48 h with few hyphae and a scarce number of inflammatory foci (Fig. 3, Table 2). All siderophore mutant strains previously tested in a neutropenic aspergillosis model showed (i) a low number of germinated conidia compared to *wt* and (ii) only discreet inflammation, even after 72 h of growth in *vivo* [4,14]. Thus, despite the limited number of lungs investigated, results obtained are in line with previously published data on mouse lung experiments [4,14]. Importantly, all the reconstituted strains showed *wt*-like inflammation and invasion values (data not shown).

## 4 Discussion

In the lung, which is the predominant site of infection for airborne fungal pathogens, AM are the first line of defense against invading pathogens such as inhaled conidia of *A. fumigatus*. Both extra- and intracellular siderophores have been shown to represent key players in the iron metabolism and consequently virulence of *A. fumigatus* [4,14]. Lack of the entire siderophore system renders *A. fumigatus* avirulent whereas lack of either extra- or intracellular siderophore biosynthesis causes partial attenuation in a murine model of pulmonary aspergillosis [4,14]. In this study we discriminated the role of intra- and extracellular siderophores during infection by comparing *A. fumigatus wt* and different siderophore-defective mutant strains in interaction with AM.

The first defense of AM is the engulfment of fungal conidia. Several mechanisms, which alter or even inhibit phagocytosis have been described; e.g. gliotoxin alters the morphology of macrophages [21,22] and decrease phagocytosis [23,24]; spore diffusates can abrogate engulfment [25]; and sialylated molecules on conidia are important ligands for phagocytosis [26]. Consistent with a previous in vitro study with RAW264.7 macrophages [15], we did not find an influence of siderophore biosynthesis on the rate of internalization of *A. fumigatus* conidia by AM ex vivo. However, the lack of extracellular siderophores (*ΔsidD* and *ΔsidF*) resulted in a substantially increased portion of killed conidia, as well as a reduced extra- and intracellular growth rate in vivo. These data indicate that siderophore-mediated iron acquisition is important for survival of *A. fumigatus* within AM. As previously shown, a deletion of *sidC* renders *A. fumigatus* more sensitive to H_2_O_2_ in vitro [14] and leads to an impaired distribution of iron within a fungal cell [27]. The individual lack of intracellular siderophores (*ΔsidC*) did not significantly impact growth and killing rates. Nevertheless, intracellular siderophores also appear to play a crucial role because the lack of extra- and intracellular siderophores (*ΔsidA*) caused a significantly higher conidial killing rate compared to the lack of extracellular siderophores only. The protective function of the siderophore system was more evident at 48 compared to 24 h after infection, which most likely explains why the siderophore system was not found to influence elimination of *A. fumigatus* by RAW264.7 macrophages 7 h after infection in vitro [15]. Nevertheless, phagocytosis of *A. fumigatus* induces transcriptional up-regulation of NADPH-oxidase subunit phox p47 and TNF-α expression in RAW264.7 macrophages in vitro, whereas the *ΔsidA* mutant does not induce this effect [15]. The latter is most likely due to the significantly increased killing rate and therefore reduced intracellular growth found here. The in vivo growth promoting functions of the siderophore system accompanies the histology: inactivation of extra- or intracellular, and in particular inactivation of both, reduced inflammation and fungal burden. The resistance to killing of *ΔsidC* conidia in vivo by AMs and the killing of *ΔsidA* conidia without up-regulating NADPH-oxidase subunit phox p47 support the hypothesis, that reactive oxygen species do not play an important role in elimination of *A. fumigatus* conidia in vivo as also indicated by other studies, which demonstrated unaltered virulence of mutants defective in oxidative stress response [28,29].

Triacetylfusarinine C has been shown to be able to extract iron in vitro from proteins binding iron with extremely high affinity, such as holotransferrin [11]. Therefore one can hypothesize that extracellular siderophores sequester iron during intracellular growth from host iron proteins. In contrast, intracellular siderophores are involved in intra-hyphal iron distribution and iron storage [27]. In agreement with the crucial role of the siderophore system in intra- and extracellular growth function siderophore biosynthetic genes such as *sidA*, *sidC*, *sidD* and *sidF* are expressed in vivo during the onset of an infection [30] and *sidD* is more prominently expressed in a macrophage cell line compared to *sidC*, but both are expressed in media containing serum [31].

Numerous studies prove the essentiality of extracellular siderophores for bacterial growth inside macrophages e.g. salicylate-derived mycobactin of *Mycobacterium tuberculosis* [32], anthrachelin of *Bacillus anthracis* [33], and 2,3-dihydroxybenzoic of *Brucella abortus* [34], but bacteria do not possess intracellular siderophores. Furthermore, treatment of macrophages with desferroxamine inhibited the intracellular conidium-to-yeast transformation of *Paracoccidioides brasiliensis* [35]. Similarly, the dimorphic fungus *Histoplasma capsulatum*, which during pathogenic growth proliferates within the macrophage phagolysosome in the yeast form, relies on siderophores during in vivo growth [36]. In contrast, the yeast *C. albicans* lacks siderophores [37]. Taken together, this study demonstrates for the first time the crucial in vivo role of extra- and intracellular siderophores in intracellular growth for a filamentous fungus.

## Figures and Tables

**Table 1:** *A. fumigatus* strains used in this study.

Strain	Genotype	Reference
ATCC4664*5*	*wt*	American Type Culture Collection
*ΔsidA*	ATCC46645, *ΔsidA::hph*	[4]
*ΔsidC*	ATCC46645, *ΔsidC::hph*	[14]
*ΔsidD*	ATCC46645, *ΔsidD::hph*	[14]
*ΔsidF*	ATCC46645, *ΔsidF::hph*	[14]
*sidA*^R^	*ΔsidA, ΔsidA::sidA*	[4]
*sidC*^C^	*ΔsidC, (p)::sidC, (p)::ptrA*	[14]
*sidD*^C^	*ΔsidD, (p)::sidD, (p)::ptrA*	[14]
*sidF*^C^	*ΔsidF, (p)::sidF, (p)::ptrA*	[14]

^R^Indicates a silently mutated version of *sidA*.

^C^Indicates the presence of an ectopically integrated, complementing allele.

(*p*) Indicates that the respective gene was transformed as a subcloned copy on a plasmid.

**Table 2:** Inflammation and fungal invasion indices of *wt* and siderophore mutant strains.

	*wt*	*ΔsidA*	*ΔsidF*	*ΔsidD*	*ΔsidC*
Inflammation index					
24 h	2	ND	1	0.5	1
48 h	3	0.5	1.5	1.5	1.5

Invasion index					
24 h	1.5	ND	1.5	1.5	1
48 h	3	1.5	2	2	1.5

For technical reasons, the number of analyzed lungs varied: two for *ΔsidF*, and three for all other strains tested.

ND = Not determined.
